# Social inequality in sexual and reproductive health in Ecuador: an analysis of gaps by levels of provincial poverty 2009–2015

**DOI:** 10.1186/s12939-019-0951-0

**Published:** 2019-06-03

**Authors:** Juan Pablo Gutiérrez, René Leyva Flores, Belkis Aracena Genao

**Affiliations:** 1 Center for Research on Policies, Population & Health, School of Medicine, National Autonomous University of Mexico (UNAM), Mexico City, Mexico; 2 Center for Research on Health Systems, National Institute of Public Health, Cuernavaca, Morelos Mexico; 3 Center of Information for Health Decisions, National Institute of Public Health, Cuernavaca, Morelos Mexico

**Keywords:** Inequality, Sexual and reproductive health, Ecuador

## Abstract

**Background:**

Adequate access to sexual and reproductive health services is associated with better results. Analyzing the differences in access and outcomes of sexual and reproductive health (SRH) by share of poverty at the regional level makes it possible to measure the magnitude of the challenge of inequity. This paper aims to estimate the magnitude of health inequality in SRH in Ecuador for the period 2009–2015.

**Methods:**

This study analyzed health inequalities in sexual and reproductive health indicators (obstetric and abortion complications, caesarean and home deliveries, adolescent fertility, and maternal mortality) for 2009 and 2015 comparing provinces in Ecuador. The absolute and relative gaps were estimated between provinces grouped by the percentage of individuals in multidimensional poverty; the slope index of inequality and the relative index of inequality were estimated as measures of gradient; and finally, the concentration index was also estimated.

**Results:**

The analysis identified that obstetric complications, abortion complications, and cesareans have tended to increase from 2009 to 2015, without relevant differences between provinces ordered by poverty. Adolescent fertility decreased in the country as well as the inequality in its distribution among provinces: the CI was − 0.046 in 2015, down from − 0.084 in 2009. Home deliveries as a ratio of total deliveries have a decreasing trend with mixed results in terms of inequality: while there is a decrease in the absolute gap from − 211.06 to 184.4 between 2009 and 2015, the concentration index increased from − 0.331 to − 0.496. Finally, the maternal mortality rate increased in the period, also with greater inequality: from an absolute gap of − 39.30 in 2019, up to − 46.7 in 2015. In the same direction, the CI went from − 0.127 to − 0.174.

**Conclusions:**

Ecuador faces major challenges in terms of both levels and inequalities in SRH outcomes and access to services. These inequalities related to poverty highlight the persistence of social inequities in the country. These health inequalities affect the wellbeing of Ecuadorian women but they are amendable. There is a need for pro-equity interventions, with stronger efforts in areas (provinces) with larger socioeconomic vulnerabilities.

## Background

Health inequities, understood as unjust and avoidable inequalities in access and health outcomes among population groups [1], has become one of the most relevant challenges in the global agenda as defined in the Sustainable Development Goals (SDG) [2]. In this regard, various analyses have shown that factors that help maintain, reproduce, and even increase social inequality converge in socially vulnerable populations [3–6].

As proposed previously, we understand health inequalities as those unavoidable differences between population groups, while health inequities are inequalities that are avoidable thus unjust [7]. In that sense, measuring health inequalities that are related to socioeconomic conditions is a measure of health inequities, that is, differences that are avoidable by human means [8].

Adequate access to sexual and reproductive health (SRH) services are important determinants of better outcomes in this area, which is both directly and indirectly central to social welfare [9]. The consequences of insufficient access to contraceptive methods, obstetric care, and sexual health counseling are seen in human and country development, for example, in higher fertility rates among adolescents, unplanned and/or unwanted pregnancies, and maternal death [10, 11].

Differences in access and outcomes associated with socioeconomic conditions can be considered unjust and avoidable, and in that sense, inequitable [12]. Analyzing the differences in indicators of access and outcomes of sexual and reproductive health by socioeconomic condition provides a measure of the magnitude of the challenge of health inequity.

Worldwide, analyses have shown that populations in conditions of socio-economic vulnerability face challenges in both health access and outcomes, which maintain and even increase social inequality [13]. Latin America and the Caribbean is one of the regions with the greatest socioeconomic inequality worldwide [14]. Analysis of the relationship between SRH and poverty finds a two-way interaction [1], that is, cause and effect generate a persistent vicious cycle between poverty and poor health conditions, especially in sexual and reproductive health (SRH) [15].

Health inequalities are results of social determinants of health. These social determinants affect health conditions, and are not homogenously distributed across population. It has been argued that these factors are particularly relevant in Latin America and the Caribbean [16].

This perspective highlights gender disparities that are reflected in in unwanted pregnancy, maternal mortality, adolescent pregnancy and unsafe abortions, whose distribution is concentrated in women with fewer economic resources and who mostly live in developing countries [11].

To change this, countries have developed different approaches to public policy, with the purpose of helping to improve people’s living conditions; however, the scope of these objectives and goals is limited by several factors that require monitoring to know their weight or influence on the results achieved.

The design of a public policy is justified insofar as it helps correct unjust and avoidable differences observed among population groups, in this case related to health indicators. As examples, we identify the inequitable provision of services and the unequal distribution of risks and harm to health, which can be linked to social disadvantages, such as low income or low educational level, among others [17].

Ecuador is a South American country with 22.9% of the population living under the income poverty line ($1.90) in 2016 [18]. Of the total population, 50.5% are women, a third of the population lives in rural areas, and 13.2% of households are female headed. Ecuador has a Gender Inequality Index of 0.385 for 2017, close to the Latin America and Caribbean average of 0.386, which ranked the country 88th in the world [19]. In terms of specific needs, adolescents have been identified as requiring improved access to sexual and reproductive services [20].

The Government of Ecuador during the period 2008–2015 implemented a set of public policies aimed at reducing extreme poverty and increasing access to health services for the most vulnerable groups, with the purpose of “reducing social, territorial, economic, environmental, and cultural inequalities” [21]. In addition, increased investment in health was observed at 3.3 percentage points of GDP (from 5.9% in 2006 to 9.2% in 2015) [22]. In this context of poverty reduction and a significant increase in public spending on health, improved access to health services and SRH conditions at the national level would be expected, as well as the reduction of inequality gaps between provinces based on their condition of poverty.

According to the World Bank estimations, the total fertility rate (TFR) for Ecuador in 2015 was 2.514, a 6.3% decrease from the 2.684 in 2009, but a higher rate than the Latin America and Caribbean average of 2.082 in 2015. Official estimations in Ecuador project a TFR around 2.0 until 2035. Moreover, there is major heterogeneity in the TFR: it was 3.9 in the province of Morona Santiago in 2015 and 2.1 in the province of Pichincha the same year. That is, the country will need about 20 years to average a TFR that has been already reached in one province.

This analysis was performed to analyze the relationship between poverty, access to services, and SRH outcomes in the period 2009 to 2015 in Ecuador. To this end, the level and trends of a set of indicators of sexual and reproductive health access at the national and provincial level were generated. The analysis compared across groups of provinces based on the percentage of households in multidimensional poverty.

## Methods

An ecological epidemiological analysis was carried out based on secondary information using standard metrics for measuring health inequalities among provinces, the first subnational level in Ecuador. The territorial organization of the Ecuadorian State establishes decentralized governments with political, administrative and financial autonomy [23]. These territorial areas, called provinces, have been grouped, according to their location, into four regions: La Sierra (Andean highlands), El Oriente (Amazon region), La Costa (Pacific lowlands), and the Galápagos Islands. The Andean highlands region is made up of the provinces of Carchi, Imbabura, Pichincha, Cotopaxi, Tungurahua y Chimborazo, Bolívar, Cañar, Azuay and Loja. The Amazon Region is composed of Sucumbíos, Napo, Pastaza, Orellana, Morona Santiago, and Zamora Chinchipe. The Pacific lowlands contains the provinces of Esmeraldas, Manabí, Los Ríos, Guayas and El Oro. The Galapagos Islands are composed of 13 main islands [24]. These provinces have enormous differences in terms of socioeconomic and health conditions [25].

According to the rules of operation, these governments have as their main function local implementation of national programs, which are financed and monitored by the central government [23].

The purpose of the analysis was to quantify the magnitude of the relationship between poverty, access to services, and SRH outcomes. To do this, it was necessary to have disaggregated information that allowed for comparisons over time and at the population level. The largest disaggregation of information, for the period studied, was at the provincial level.

As measures of gaps, we estimated absolute (the magnitude of the difference in health between subgroups) and relative (the ratio of differences in health indicators between subgroups). For measures of gradient, the slope index of inequality (SII) and the relative index of inequality (RII). Finally, the Wagstaff adjusted Concentration Index, as described below.

All analysis used as the socioeconomic indicator (stratifier), the multidimensional poverty index (MPI) of Ecuador, that is, the official measure of poverty in the country. The MPI comprises 4 equally weighted dimensions (education; social security and employment; health, nutrition and water; and environment, habitat and dwelling) and 12 indicators. Education indicators are related to school attendance and schooling achievements. Social security measures pension contribution, while employment measures unemployment and child/adolescent work. Health, nutrition and water uses income and access to water. Finally, habitat and environment uses access to sanitation and trash collection, while dwelling conditions uses overcrowding and dwelling deficit. An individual is considered as poor if they are identified as deprived in at least one third of the indicators [26].

### Analyzed indicators

In the development agenda, signed by most countries in the world in the year 2000, it was agreed to improve maternal health, which meant a reduction of 75% in the maternal mortality rate between 1990 and 2015 [8]. Reducing the maternal mortality rate necessitated increasing the quality of the obstetric health care. In underdeveloped and developing countries, the aim of increasing the quality of obstetrics care is attempted through increasing hospital coverage of obstetric emergency [27, 28]. Similarly, adolescent pregnancies are a global priority, due to the social impact and the risk they represent for adolescent health. As such, the indicators of home birth, obstetric complications, and fertility in adolescents, are elements affecting the frequency of maternal mortality. For these reasons and due to the availability of information for all provinces in the period analyzed, the indicators included in the present investigation were selected.

### Obstetric complications ratio

This addresses the morbidity that occurs during pregnancy, delivery, and puerperium, and is defined as the ratio of women who received care for gynecological and obstetric events, identified through ICD-10 codes (Codes: 000–099) among total live births in the same period, multiplied by one thousand.

Post-abortion complications ratio is a subcategory of obstetric complications indicator that considers only women with abortion discharges.

Ratio of births attended at home. Number of births attended at home per 1000 live births as an indicator of access to reproductive health services. Evidence has suggested that compared to hospital deliveries, there are suboptimal outcomes for both mother and child at home deliveries [29, 30].

### Percentage of births by caesarean section

Indicator of structural quality of reproductive health services, calculated as the number of births by Caesarean section over the number of deliveries attended in the same period.

### Age-specific fertility rate 15–19 (adolescent pregnancy) (APFR 15–19)

This is the age-specific fertility rate for the group of women aged 15 to 19 years. The indicator was calculated as the quotient of the number of live births from women in the group of 15 to 19, in a given geographical area in a year, by every thousand women in that age group. The legal age for marriage in Ecuador is 18 as of 2015, but data from 2017 reports that 22% of Ecuadorian girls are married before that age [31]. Early childbearing negatively affects both women’s and children’s wellbeing [32, 33].

### Maternal mortality ratio (MMR)

This is the main outcome indicator of sexual and reproductive health care, and a central indicator in the Sustainable Development Goals. It is calculated as the quotient of the number of maternal deaths in women from 15 to 49 selecting ICD-10 codes O00 to O99, excluding codes O96 and O97 (late maternal deaths) according to the standard procedure in Ecuador for identifying maternal deaths, over the total number of births, multiplied by 100,000.

### Information sources

Public secondary information was used from the National Institute of Statistics and Censuses (Instituto Nacional de Estadística y Censos, INEC), the National Secretariat for Planning and Development (Secretaria Nacional de Planificación y Desarrollo, SENPLADES), the Coordinating Ministry for Social Development (Ministerio Coordinador de Desarrollo Social, MCDS) and the Ministry of Public Health of Ecuador (Ministerio de Salud Pública del Ecuador, MSP). In particular, information was extracted from the following sources that are collected by the National Institute of Statistics and Censuses (INEC) of Ecuador:

- Statistical yearbooks of births and deaths published by INEC (http://www.ecuadorencifras.gob.ec/anuario-de-nacimientos-y-defunciones/).
- National Survey of Health and Nutrition 2012 (Encuesta Nacional de Salud y Nutrición, ENSANUT) (http://www.ecuadorencifras.gob.ec/salud-salud-reproductiva-y-nutricion/).
- Databases of live births and deaths. (http://www.ecuadorencifras.gob.ec/nacimientos_y_defunciones/).
- Multidimensional poverty index. (http://www.ecuadorencifras.gob.ec/pobreza-multidimensional/).
- Database of hospital discharges (http://www.ecuadorencifras.gob.ec/estadisticas-de-camas-y-egresos-hospitalarios-bases-de-datos/).

### Analysis of trends in indicators

To explore the behavior of the indicators over time, we first did a descriptive analysis of their values between 2008 and 2015 for Ecuador as a whole, calculating the value for each year according to the previous definitions. This analysis made it possible to identify indicators that are overall improving, worsening or remaining unchanged during the period.

### Analysis of health inequalities

To generate an intra-country analyses of health inequalities in Ecuador, we compared provinces in the country by the different indicators and using the different metrics of inequalities.

For the gap analysis (absolute and relative), we first ordered provinces by the percentage of households living in poverty (using the multidimensional measure of poverty) and then divided them into 5 equal size groups (quintiles) [34]. For each quintile, we estimated the different indicators as the weighted averages of the indicator value in the provinces in that quintile:

$$ {SRHI}_q=\sum \limits_i^n{SRHI}_i\ast \frac{Pop_i}{POP_q} $$

where *SRHI*_*q*_ is the indicator for the quintile (1 to 5), *SRHI*_*i*_ is the value of the indicator in province *i* and $\frac{Pop_i}{POP}$ measures the relative size of the population of each province related to the population of all province in that quintile.

For the gap analysis, the absolute gap is the difference in *SRHI*_*q*_ between quintiles 5 and 1, while the relative gap is the ratio between the same quintiles:

$$ AbsoluteGap={SRHI}_{q5}-{SRHI}_{q1} $$

$$ RelativeGap=\frac{SRHI_{q5}}{SRHI_{q1}} $$

The absolute gap will result in a positive value if the indicator is pro-rich (higher value for the quintile 5) and negative if pro-poor. The relative gap will be > 1 for pro-rich indicators and < 1 for pro-poor indicators.

We estimated the slope index of inequality (SII) using linear regression models where the dependent variable is the value of the indicator at the province level and the independent variable is the ridit for the percentage of households living in poverty by province [34–36]:

$$ {SRHI}_i=\alpha +\beta {Ridit}_i+\varepsilon $$

where *Ridit*_*i*_ is the ridit score of the share of poverty in each province, *α* is the intercept or the level of SRHI in absence of poverty, *β* is the SII and *ε* is the term of error. The SII measures the difference in the outcome between the provinces in the extremes of the actual distribution. For this case, negative values of the SII indicates that the SRHI is higher at the poorest province compared with the richest province. The SII is an absolute measure that is sensitive to changes in the level of the health indicator in the population even if differences among provinces remain constant.

Thus, we also estimated the relative index of inequality that is the ratio of the coefficients in the estimated regression, equivalent to the ratio on the SRHI at the extremes of the poverty distribution, that is the difference of zero poverty versus 100% poverty [35, 37]:

$$ RII=\frac{\widehat{SRHI_0}}{\ \widehat{SRHI_1}}=\frac{\alpha }{\ \alpha +\beta } $$

We finally estimated the Concentration Index, which is derived from a concentration curve of the health indicator and the population ordered by a socioeconomic indicator,in this case, provinces ordered by share of poverty. The CI is generally defined as twice the area between the concentration curve and the line of equality (the 45-degree line), and ranges from − 1 to 1. For CI, the zero value is an indication of absence of inequality, while for this analysis, positive values indicates pro-poor indicators, that is, indicators that present higher values for provinces with a larger share of poor households and, in the opposite, negative CI values identify pro-rich indicators [34, 35, 38].

We did all the analyses in Stata 15.

## Results

### Analysis of national trends

**Fig. 1:**
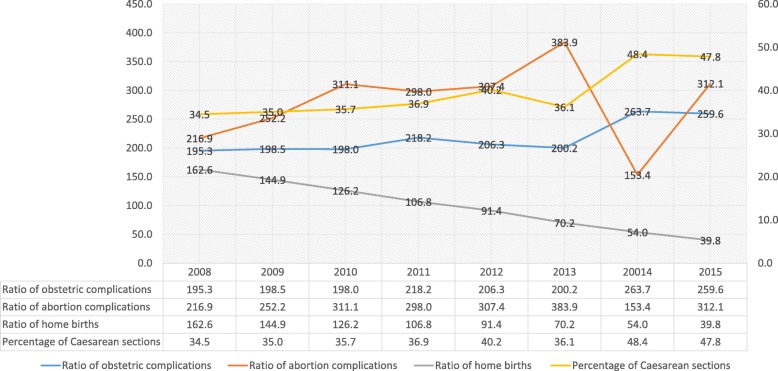
Trends in Access to health services by SRH needs, Ecuador 2008-2015

Figure 1 shows the trends between 2008 and 2015 for the indicators related to care for SRH needs in health services. It is seen that, while deliveries attended at home tended to decrease, a favorable result, there are increases in the percentage of births by Caesarean section (unfavorable results) and obstetric complications in general, and specifically post-abortion complications.

**Fig. 2:**
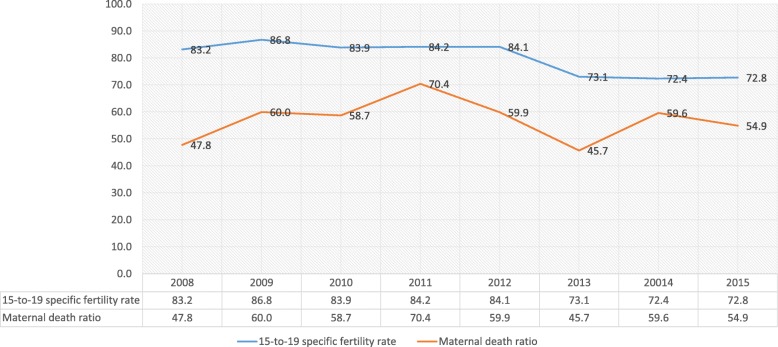
Trends in SRH Results, Ecuador 2008-2015

On the other hand, Fig. 2 shows the indicators of results, observing that while there is a favorable decrease of 12.5% in the specific fertility rate of ages 15 to 19 between 2008 and 2015, the maternal mortality ratio increased in 14% in the same period.

### Analysis of inequalities between provinces

The results from the analysis of inequalities are reported in Table 1 by indicator and year (2009 and 2015).

**Table 1:** Measures of inequality in selected indicators of sexual and reproductive health in Ecuador for 2009 and 2015, stratified by multidimensional poverty

Measure	Year	Ratio of obstetric complications	Ratio of abortion complications	Ratio of home births	ASFR 15–19^a^	MMR^b^	Caesarean births
Absolute Gap	2009	56.11	NS	−211.06	− 33.87	−39.30	NS
	2015	NS	NS	−184.4	−7.61	−46.7	27.02
Relative Gap	2009	1.32	0.65	0.17	0.70	0.53	1.23
	2015	0.90	NS	0.06	0.90	0.48	2.05
SII	2009	106.25	NS	− 258.21	−44.69	−45.96	NS
	2015	NS	NS	− 121.39	−20.32	−58.64	NS
RII	2009	1.719	NS	0.007	0.588	0.440	NS
	2015	NS	NS	0.208	0.755	0.304	NS
CI	2009	0.086	NS	−0.331	−0.084	−0.127	NS
	2015	NS	NS	−0.496	−0.046	− 0.174	NS

^a^ASFR: age-specific fertility rate, ^b^ MMR: maternal death ratio, *SII* slope index of inequality, *RII* relative index of inequality, *CI* concentration index, *NS* not significant

As shown in the table, there are two sets of indicators by results: indicators that have clear patterns of inequalities affecting poorer provinces (home births, adolescent fertility rate and MMR), and indicators related to the delivery processes that seem not to differ by poverty level by 2015 (obstetric and abortion complications, and Caesarean deliveries).

By 2009, obstetric complications were higher in provinces with a lower share of poverty, with a concentration index of 0.086 suggesting a slight inequality in the distribution of this type of complications that were more frequent for those women living in less poor provinces. Nevertheless, by 2015 there were no significant differences in this indicator by provincial poverty level.

The pattern for abortion complications suggests an equal distribution by province, regardless of the share of poverty. That is, the only significant result was a relative gap of 0.65 in 2009 –suggesting a larger ratio for provinces with a lower share of poverty—but by 2015, there are no significant differences among province, neither for gaps, nor for gradient measure or concentration index.

Home deliveries are clearly concentrated in provinces with a larger share of poverty both in 2009 and 2015. While the gap and gradient measures suggest an improvement in the inequality of this indicator from 2009 to 2015 (the absolute gap drops from − 211.06 to − 184.4, and the RII that was 0.007 in 2009 is 0.208 by 2015), the concentration index suggests an increase in the inequality, as it goes from − 0.331 in 2009 to − 0.496, that is, a larger share of home deliveries at provinces with a larger share of poverty.

As for the age-specific fertility rate 15–19, the results highlight an unequal distribution across provinces, with a higher rate in provinces that also have a larger share of poverty, which is consistent for gap, gradient, and concentration measures. Nevertheless, inequality in this indicator decreased from 2009 to 2015: the absolute gap falls from − 33.87 in 2009 to − 7.61 in 2015, and the same pattern is reflected in the relative gap that gets closer to one, from 0.70 in 2009 to 0.90 by 2015. The SII decreased from − 44.69 in 2009 to − 20.32 in 2015, so the difference between provinces in the extremes of poverty share shrinks in that period. As the RII approaches one (no differences among provinces) moving from 0.588 in 2009 to 0.755 in 2015, the CI dropped from − 0.084 to − 0.046 in the same period.

The opposite occurred for the maternal mortality ratio: the absolute gap increased from − 36.30 to − 46.7, that is an additional 10.4 more maternal deaths per 100,000 live births in the provinces with the larger share of poverty compared to those provinces with the lower share of poverty, with a slight change in the relative gap from 0.53 to 0.48. The SII increased the difference among provinces from − 45.96 to − 58.64, that is 12.68 more maternal deaths per 100,000 live births. The concentration index increased from − 0.127 to − 0.174.

Finally, for Caesarean births, there are no significant results for gradient nor concentration measures. The relative gap increased from 1.23 to 2.05, suggesting a larger proportion of Caesarean deliveries among provinces with a lower share of poverty compared to those with the largest share of poverty.

**Fig. 3:**
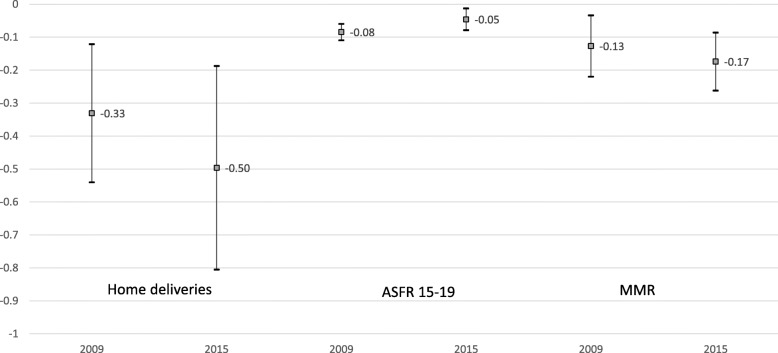
Concentration index for home deliveries, ASFR 15-19 & MMR in Ecuador, 2009 & 2015

In Fig. 3, we see the concentration index with the 95% confidence intervals for the 3 variables with significant results both in 2009 and 2015. As previously mentioned, values for the CI in these 3 variables suggest that inequality increased from 2009 to 2015 regarding the ratio of home deliveries as well as the maternal mortality rate, whereas it decreased for the age-specific fertility rate 15–19. Nevertheless, in the 3 cases, the 95% CI for both years overlap.

## Discussion

There is a complex relationship between poverty and access to health services and health outcomes, also affecting SRH. In general, there is a direct relationship between better socioeconomic status and better SRH outcomes [39], but is also clear that this is related to the specific context. Nevertheless, worldwide maternal deaths are disproportionally concentrated in low-income countries and among rural and poor women [40].

The results in this paper highlight a scenario with challenges for Ecuador, both regarding sexual and reproductive health services and outcomes levels, but also in terms of social inequalities and SRH (Table 2). Previous research has already discussed how access to health services in Ecuador is affected by socioeconomic level and thus the need to increase access for those socioeconomically vulnerable [41].

**Table 2:** Ecuador: Scenarios of SRH and social inequality among provinces 2009 to 2015

		Social Inequality in Health	
		Tapering	Widening
Average population health trend	Improving	Best result • Adolescent fertility rate • *Ratio of home deliveries*	*Improvement with inequality*
	Worsening	*Worsening with protection* • Ratio of obstetric complications Ratio of abortion complications	Worst result • *Percentage of Caesarean sections* • Maternal mortality ratio

While for some indicators there is a clear overall improvement with a reduction of inequality (fertility rate among adolescents and ratio of home births), for other relevant indicators, such as the MMR, overall country levels increased between 2009 and 2015 with widening inequality between provinces (worst result).

During the period from 2009 to 2015, Ecuador has presented advances in increasing access and results for SRH indicators. However, the gaps associated with poverty persist, which are reflected in lower access and unfavorable results for populations with higher levels of poverty. This suggests that increasing general access may not reduce inequality if other barriers to access (economic, cultural, etc.) persist [42].

Maternal mortality remains a challenge for the country. This SRH indicator reports timid achievements, both on average and in terms of its distribution in the population, affecting mainly the population in poverty.

A recent analysis of inequalities in MMR in Ecuador have already reported differences at the province level but using different socioeconomic indicators [25]. Results reported here are consistent in that they identify SII and RII measures indicating larger MMR for more socioeconomically vulnerable provinces. While the reported SII using the MPI in this paper is − 45.96, the estimation using gross domestic product is − 28.8 [43]. This paper contributes not only by using Ecuador’s official measure of poverty that also follows international recommendations in using multidimensional measures, but also by including the concentration index and a set of indicators that allow for a broader picture.

The fertility rate among adolescents declined significantly at the national level, with a greater reduction in the provinces with the highest concentration of poverty. As has been reported in the region, increased school attendance could explain this result [44].

Home births were reduced at the national level; however, the gap along provinces’ socio-economic characteristics persists. The provinces with the highest proportion of the population in poverty have the highest frequency of home birth. In other settings, it has been reported that home deliveries are a consequence of lack of access to health services, that is, it is not by choice [45, 46]. Home birth represents one of the greatest challenges to the goal of reducing maternal mortality in developing countries. Its high frequency in these countries is related to geographic access problems [46]. In this sense, we identify that it is not home delivery care per se that constitutes a risk factor, but rather accessibility to care units that can resolve problems before an obstetric emergency. Therefore, home birth care in developing countries is an indicator of inequity, responding to restricted opportunities for hospital care, and not a voluntary decision.

Obstetric complications, their frequency, and type may depend on the sociodemographic conditions of the affected women; however, the outcomes and sequelae could be more related to the opportunity to access quality health services. In 2015, complications were more frequent in provinces with a lower concentration of poverty, representing a change compared to 2009. This situation could be because the provinces that have the hospitals with the greatest resolution capacity receive referrals from other provinces for their care. Other studies have already reported that seeking care for obstetric complications is related to socioeconomic conditions [47].

Caesarean births represent an important window of opportunity in the country. This indicator increases over time, in addition to reflecting differences among quintiles, showing the provinces with the lowest concentration of poverty reporting the highest frequency of Caesarean section, in contrast to the rest of the indicators. This indicator is related, as in other developing countries, to doctors’ attitudes on pregnancy resolution at the hospital level. Turkey and Mexico are the OECD countries where almost 50 out of every 100 live births are via Caesarean [48], unlike Sweden and Holland where 16 out of every 100 live births are via Caesarean section. In an analysis of Caesarean sections in 150 countries, between 1990 and 2014, the largest absolute increase occurred in Latin America and the Caribbean (19.4%, from 22.8 to 42.2%) (49). Reasons indicated for this increase are multifactorial and not well-understood. Changes in maternal characteristics and professional practice styles, increasing malpractice pressure, as well as economic, organizational, social, and cultural factors have all been involved in this trend (50). Other elements that have been described to understand the increase in Caesarean sections include inequities in the use of the procedure, not only between countries but also within countries, and the costs that unnecessary Caesarean sections impose on financially stretched health systems (51). However, in Ecuador this situation may be more serious, and represent a clear indicator of social inequity in health, since the increase is concentrated in those provinces with the highest proportion of the population in poverty. A recent analysis of inequalities in MMR in Ecuador have already reported differences at the province level but using different socioeconomic indicators [25]. Results reported here are consistent in that they identify SII and RII measures indicating larger MMR for more socioeconomically vulnerable provinces. While the reported SII using the MPI in this paper is − 45.96, the estimation using gross domestic product is − 28.8 [43].

This paper contributes not only by using Ecuador’s official measure of poverty that also follows international recommendations in using multidimensional measures, but also by including the concentration index and a set of indicators that allow for a broader picture.

Other health inequalities have been described for Ecuador; for example, measles vaccination coverage has been found to be inversely related to poverty, with a SII of 10.6% points difference on coverage between the canton with the highest socioeconomic level and the canton with the lowest socioeconomic level, and a RII of 1.12 [43], measures that are consistent to those for SRHI reported in this paper.

The inequalities mentioned express inequities, insofar as they are indicators that the health system can change through interventions, since it is the country’s health system itself that should respond similarly to different population groups, regardless of their socioeconomic status. Moreover, the concentration of households in poverty is not independent of the distribution of other relevant stratifiers, such as indigenous population, as the provinces with the highest concentration of poverty are those with the highest percentage of indigenous population.

There are some limitations for this analysis. Firstly, it is based on information generated from administrative systems, which presents the challenges of information systems in low- and middle-income countries. In general, it is expected that there will be greater under-registration in the areas of greatest poverty, so the gaps that are reported could be even greater. Improvements in the quality reporting of indicators, at the population level, could be useful to identify differentials even within the provinces. Nevertheless, using different years and indicators makes it possible to check the consistency of the results.

Another limitation is the ecological fallacy, that is, associations among provinces do not necessarily reflect association among individuals. Nevertheless, for this analysis what is relevant is the fact that access and outcomes are worse for those living in provinces with a larger share of poverty.

While these results refer to an analysis specific to Ecuador, they point to the global importance of addressing social inequalities in general, and particularly in health. Research in Latin America and the Caribbean has already discussed the relevance of social inequalities in terms of access to health services [48].

While closing gaps between populations has been established as one of the Sustainable Development Goals, it is necessary to examine in a transversal logic, in which poverty reduction and the improvement of health conditions in the logic of better health are implemented while explicitly aiming to close socio-economic and health gaps.

Decisions focused on caring only for SRH indicators, without considering socioeconomic differences between populations, may appear successful by impacting that level; however, these advances happen at the cost of leaving population groups behind.

## Conclusions

The present study aimed to contribute to generating evidence on the relationship between public policies that seek to improve the life and health conditions of populations. Social inequalities in SRH are relevant but also amendable. Interventions must use a pro-equity approach, with stronger efforts on areas (provinces) with larger socioeconomic vulnerabilities, acknowledging the important role of the social determinants of health.
